# Editorial: listening for congestion in heart failure

**DOI:** 10.1093/ehjdh/ztag096

**Published:** 2026-06-13

**Authors:** Fabian Kerwagen, Maximilian Bauser, Stefan Störk

**Affiliations:** Department of Internal Medicine I, Cardiology, University Hospital Würzburg, Oberdürrbacherstrasse 6, 97080 Würzburg, Germany; Department of Clinical Research and Epidemiology, Comprehensive Heart Failure Center, University Hospital Würzburg, Am Schwarzenberg 15, 97080 Würzburg, Germany; Department of Internal Medicine I, Cardiology, University Hospital Würzburg, Oberdürrbacherstrasse 6, 97080 Würzburg, Germany; Department of Clinical Research and Epidemiology, Comprehensive Heart Failure Center, University Hospital Würzburg, Am Schwarzenberg 15, 97080 Würzburg, Germany; Department of Internal Medicine I, Cardiology, University Hospital Würzburg, Oberdürrbacherstrasse 6, 97080 Würzburg, Germany; Department of Clinical Research and Epidemiology, Comprehensive Heart Failure Center, University Hospital Würzburg, Am Schwarzenberg 15, 97080 Würzburg, Germany

## Abstract

**Graphical Abstract:**
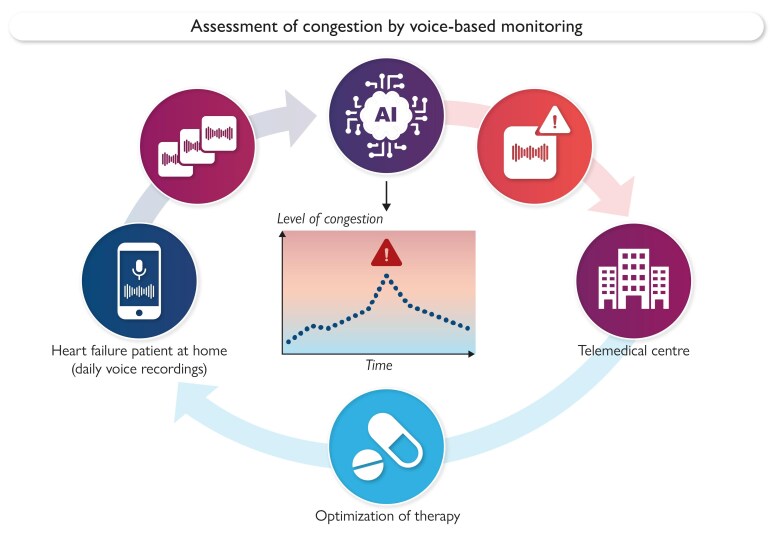

**This editorial refers to ‘A pilot study on AI-based voice analysis for monitoring patients hospitalized with acute decompensated heart failure’, by L. Riehle *et al*. https://doi.org/10.1093/ehjdh/ztag052.**

Monitoring congestion in heart failure remains one of the central challenges in cardiovascular medicine. Despite decades of research, commonly used tools such as symptom assessment and body weight provide only limited sensitivity for detecting changes in congestion, while more advanced approaches—such as invasive haemodynamic monitoring—are effective but invasive and not easily scalable.^1–4^ This gap has driven increasing interest in digital biomarkers that are non-invasive, continuous, and widely accessible. The idea that the human voice, an easily obtainable and ubiquitous signal, may carry clinically meaningful information about cardiopulmonary status represents a potentially important advance in digital cardiovascular monitoring. This concept also resonates with everyday clinical experience: clinicians, nurses and lay people alike often recognize when a patient ‘does not sound well’ suggesting that the health status is, at least in part, encoded in the human voice. As such, if the voice indeed contains a reproducible acoustic signature of worsening heart failure, it could offer a window into cardiopulmonary status. Building on early proof-of-concept studies,^5,6^ Riehle *et al.*^7^ report on a highly appealing concept: the use of voice and breathing analysis as a potential marker of clinical status in patients with acute decompensated heart failure.

## The innovative concept

In a prospective pilot across two centres from Germany and the USA, the authors analysed voice samples from 79 patients with acute decompensated heart failure and obtained daily voice recordings until discharge. Baseline N-terminal pro-B-type natriuretic peptide (NT-proBNP) levels were high with 9400 pg/mL, and patients lost about 5 kg of body weight over a mean hospitalization period of 7 days. A machine-learning model, trained on voice and breathing features extracted from sustained vowels, distinguished admission and discharge states with reasonable accuracy (F1 score 0.83). Model predictions allowed to generate trajectories that aligned with the clinical course during hospitalization. Despite the limited size of the cohort, the observed dynamic pattern suggested that vocal signals convey information about the evolving cardiopulmonary status beyond static ‘wet’ and ‘dry’ snapshots.

## Defining what the voice really reflects: advancements and limitations

The investigators moved from cross-sectional sampling to serial monitoring, capturing a dynamic portrait of decompensation and recompensation. The gradual shift of model outputs across intervening days lends biological plausibility by paralleling decongestion and stabilization. Nevertheless, the fundamental issue pertains to what the model actually detects. It was trained to discriminate phases of hospitalization, not congestion itself. In the absence of concurrent objective markers, such as serial natriuretic peptides, haemodynamic data, or imaging, it is not possible to ascertain whether the algorithm detects congestion-related changes potentially arising from pulmonary fluid, laryngeal oedema or altered respiratory dynamics. Importantly, the timing of hospital admission and discharge often depends on multiple factors beyond congestion alone. For instance, comorbid conditions or complications independent of the congested state (e.g. acute infection), as well as non-medical factors (e.g. the availability of adequate home care) may influence the day of discharge, thereby potentially confounding the model’s predictions.

The authors reported that model performance remained robust irrespective of significant weight loss during hospitalization. This finding is intriguing, but challenging to interpret. The voice may pick up congestion missed by scales, but it may equally indicate that the classifier responds to a different signal of convalescence. Future studies should therefore aim to better understand the pathophysiological mechanisms underlying voice changes.^8^ Furthermore, as discussed by the authors, a potential selection bias might apply: approximately one quarter of enrolled patients were excluded, including those with clinical deterioration or non-linear trajectories of body weight (i.e. higher weight at discharge compared with admission). Whereas in these patients a congestion monitoring tool would be most clinically valuable, their exclusion likely simplifies the classification task by focusing on monotonic improvement and may inflate performance estimates.

## Implications for practice and research

Unlike more established biomarker modalities, voice-based approaches present unique challenges. As voice is inherently linked to language, linguistic variability is a critical factor, underscoring the importance of multilingual datasets and validation across diverse populations. In this regard, the authors should be commended for including recordings in two languages, providing an important step towards addressing linguistic variation in voice-based biomarker research. More importantly, there is currently no universally accepted definition of a ‘vocal biomarker’, and the methodological approaches to voice acquisition, feature extraction, and analysis are inconsistent.^6^ Therefore, issues of standardization and harmonization represent key challenges for this young field.^6^ Finally, voice data are inherently personal and potentially identifiable, giving rise to specific ethical, legal, and social implications. Addressing these considerations will be essential for the responsible development and implementation of voice-based technologies in clinical practice.^6^

Notwithstanding these caveats, the attraction of voice analysis is clear. Remote haemodynamic monitoring and structured telemedicine programmes have improved outcomes, but they require implants, infrastructure, or intensive oversight.^9,10^ To advance the field of vocal biomarkers, at least three elements are needed going forward: (i) co-investigation with validated congestion parameters, (ii) a clearer pathophysiological understanding including multidimensional voice assessment in patients with acute heart failure to strengthen confidence in voice-based monitoring of congestion, and (iii) clarification of the prognostic relevance of vocal biomarkers (e.g. prediction of future worsening heart failure events). Ultimately, the key question is how well these approaches translate into clinical care. Will voice-based monitoring enable early detection of decompensation and can it effectively be integrated into remote patient monitoring frameworks? The promise is compelling: a scalable, patient-friendly tool that could extend monitoring approaches provided that we can demonstrate that what the algorithm ‘hears’ is indeed clinically actionable.
